# Comparative Evaluation of Condylar Guidance Obtained by Digital Panoramic Radiographic Images Using Two Different Interocclusal Recording Material During Jaw Relation in Completely Edentulous Patients: An In Vivo Study

**DOI:** 10.7759/cureus.70519

**Published:** 2024-09-30

**Authors:** Khushbu Doshi, Seema R Kambala, Surekha A Dubey, Pragati Agarwal, Dhanashree A Minase, Pooja Chitlange

**Affiliations:** 1 Department of Prosthodontics, Sharad Pawar Dental College and Hospital, Wardha, IND; 2 Department of Prosthodontics, Datta Meghe Institute of Higher Education and Research, Wardha, IND

**Keywords:** horizontal condylar inclination, interocclusal record, jaw relation, opg, programming, semi adjustable articulator

## Abstract

Objective

To compare the condylar guidance value obtained during jaw relation by two different interocclusal recording materials to that obtained from OPG (Orthopantomogram).

Materials and methods

Two different interocclusal recording materials were used, namely bite registration wax (reinforced wax, by MAARC Dental Company) and bite registration paste (jet bite, coltene) for interocclusal records during jaw relations. An OPG is shot prior to the treatment, and horizontal condylar guidance (HCG) is traced joining the bony landmarks in a completely edentulous patient seeking complete denture treatment. All the conventional steps for complete denture fabrication up to jaw relation are performed. A facebow transfer is done on a Hanau H2 articulator, and height tracers (extra oral tracing devices) are attached. After satisfactory training, the tracing is performed by the patient, and the interocclusal records are obtained. The articulator is programmed to obtain the value of HCG using protrusive records, which are then compared to the value obtained from OPG. Statistics were carried out to determine angles. Followed by statistical analysis with Pearson correlation to analyze the results (α = 0.01).

Results

A substantial relationship was discovered between the horizontal condylar inclination between two interocclusal recording materials and the corresponding OPG for both the right (p = 0.0015) and left (p = 0.0007) sides.

Conclusion

The condylar guidance obtained with jet bite was closer than the condylar guidance value obtained with reinforced wax when compared with the horizontal condylar inclination obtained in OPG by tracing with a mean difference of 10° for polyvinyl siloxane and 20° for reinforced wax in seven subjects evaluated.

## Introduction

Achieving proper occlusal balance and maintaining temporomandibular joint (TMJ) health in completely edentulous patients often hinges on obtaining an accurate interocclusal record. Various materials can be used to create such records. These materials should possess properties that allow for precise reproduction of the patient's occlusal relationship and jaw movements. Options include traditional materials like wax or acrylic resin, and plaster, as well as modern alternatives such as polyvinyl siloxane or polyether. Each material has its advantages and may be chosen based on factors like ease of use, accuracy, and patient comfort. Selecting the most appropriate material for interocclusal records is crucial to ensuring the successful fabrication of well-fitting dentures and minimizing the risk of TMJ-related complications. A semi-adjustable articulator commonly used in prosthodontics may simulate TMJ anatomy and mandibular movements. Thus, it functions like a patient in the absence of the patient [1]. Transferring the interocclusal records to the articulator is a vital step for manufacturing the dentures; thus, it must be accomplished accurately to decrease the clinical appointment time and also to avoid challenges for indubitably seeking the treatment [2].

Condylar guidance (CG) is described as mandibular guidance generated by the condyle and articular disc traversing the contour of the articular eminence or the mechanical form located in the posterior region of an articulator that controls the movement of its mobile member, GTP10 [3]. Two types of condylar inclination are present in the semi-adjustable articulator: the lateral condylar inclinations (LCI) and the horizontal condylar inclinations (HCI). HCI is often obtained with protrusive interocclusal records, and the LCI can be estimated using Hanau’s formula or with individual lateral records [4]. The HCI in an articulator represents the anatomic slopes of articular eminences. Lateral and protrusive interocclusal records are used to perform this programming [5]. The present study investigates the materials for their accuracy by comparing the CG obtained from orthopantomograms (OPG). According to the literature, the tomographs, OPG, and lateral cephalograms are used for recording condylar guidance. Based on studies, radiographic methods are more accurate at recording condylar guidance than other approaches [6,7]. This is due to the fact that the presence of a uniform skeletal landmark is comparable to clinical procedures with regard to its consistency [1]. The main aim of our study is to assess the precision of interocclusal recording materials at the jaw relation stage with bite registration wax and bite registration paste material and compare those values obtained from OPG for the CG.

## Materials and methods

The study was conducted on patients reporting to the Department of Prosthodontics and Crown and Bridge, Sharad Pawar Dental College and Hospital, Sawangi (Meghe), Wardha. The completely edentulous patient seeking complete dentures and having good neurological muscle coordination, which can produce great tracing on border movements of the mandible, was selected for the study. According to the sample size, seven subjects were evaluated as per inclusion and exclusive criteria. The patients lacking neuromuscular coordination and those who were not willing to participate were excluded from the study. Only those who provided us with written informed consent were considered for participation. After that, the following procedure has been performed. First, the individual was referred to the Department of Oral Medicine and Radiology for orthopantomography. There the radiograph was shot at a fixed distance in a Planmeca radiographic machine at 70 kV (kilovoltage).

**Figure 1:**
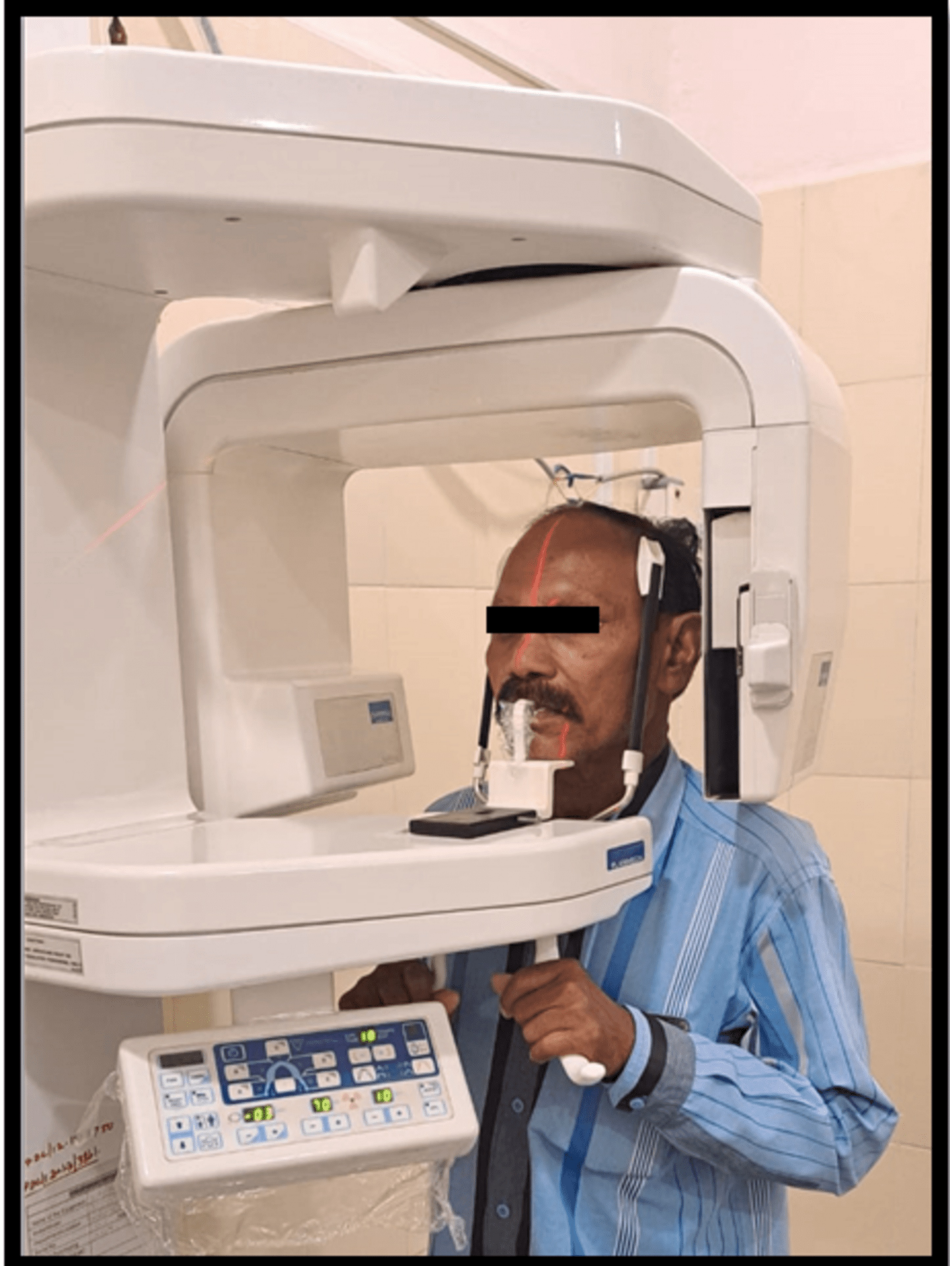
Position of head for shooting OPG OPG: Orthopantomogram

The ad positioner was used to set individuals in the machine, and cassettes were loaded. The standardized head position was made with the help of the grid positioning light of the radiograph machine to position the patient's head (Figure 1). The grid is designed in such a way that it is parallel to the Frankfort horizontal plane (FHP) of an individual. The placement of the position of the head is in an anteroposterior direction based on the manufacturer's adjustment scale and with the grid line. The head position was confirmed with the machine as the tip of the nose is to be focused as the crossing point of the light. The sagittal plane was vertically aligned with the mid-nasal and anterior nasal spines as the midline. The increased contrast between the two positions, open and closed mouth, the radiographs were exposed to at 70 Kv and 68 Kv at 10 mA. All the radiographs were taken by the same staff member at a specific duration, using the same panoramic radiography apparatus. Each individual underwent an OPG with the FHP and upper border of the radiograph parallel to the floor. As radiographic recorders in circular rotation, the upper border of the radiograph is parallel to the FHP image.

**Figure 2:**
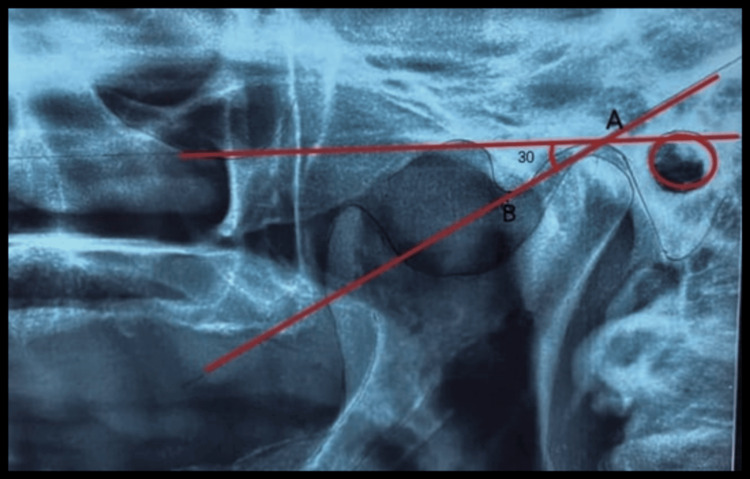
Tracing on OPG. Point A is the most superior point of curvature, and Point B is the most inferior point of curvature. The horizontal line represents the FH plane. Angle measured is 30°. OPG: Orthopantomogram, FH: Frankfort Horizontal Plane

Once a hard copy of the OPG was obtained, transparent tracing paper was attached to it, and tracings were made along slopes of articular eminence over the parchment paper. A parallel horizontal reference line was drawn on the radiograph's upper border, contacting and marking the curvature of the most superior and inferior points. Traced the outline of each curvature as well as of the FH reference plane. Tracing revealed a line connecting the most superior and inferior points of curvature, which represented the mean curvature line. Also, the mean curvature line represents this two-point connecting in a straight line. The intersection of the horizontal reference line and mean curvature line makes an angle, which is measured, that gives the CG values, which are recorded for both the right and left sides. This data is documented for all subjects (Figure 2). Then clinically, all the steps for making a complete denture were followed, and a tentative jaw relation was recorded. Facebow transfer was done using a Hanau spring bow; the upper cast was oriented to the articulator. A static record for centric relation was made with alu wax as an interocclusal recording material.

**Figure 3:**
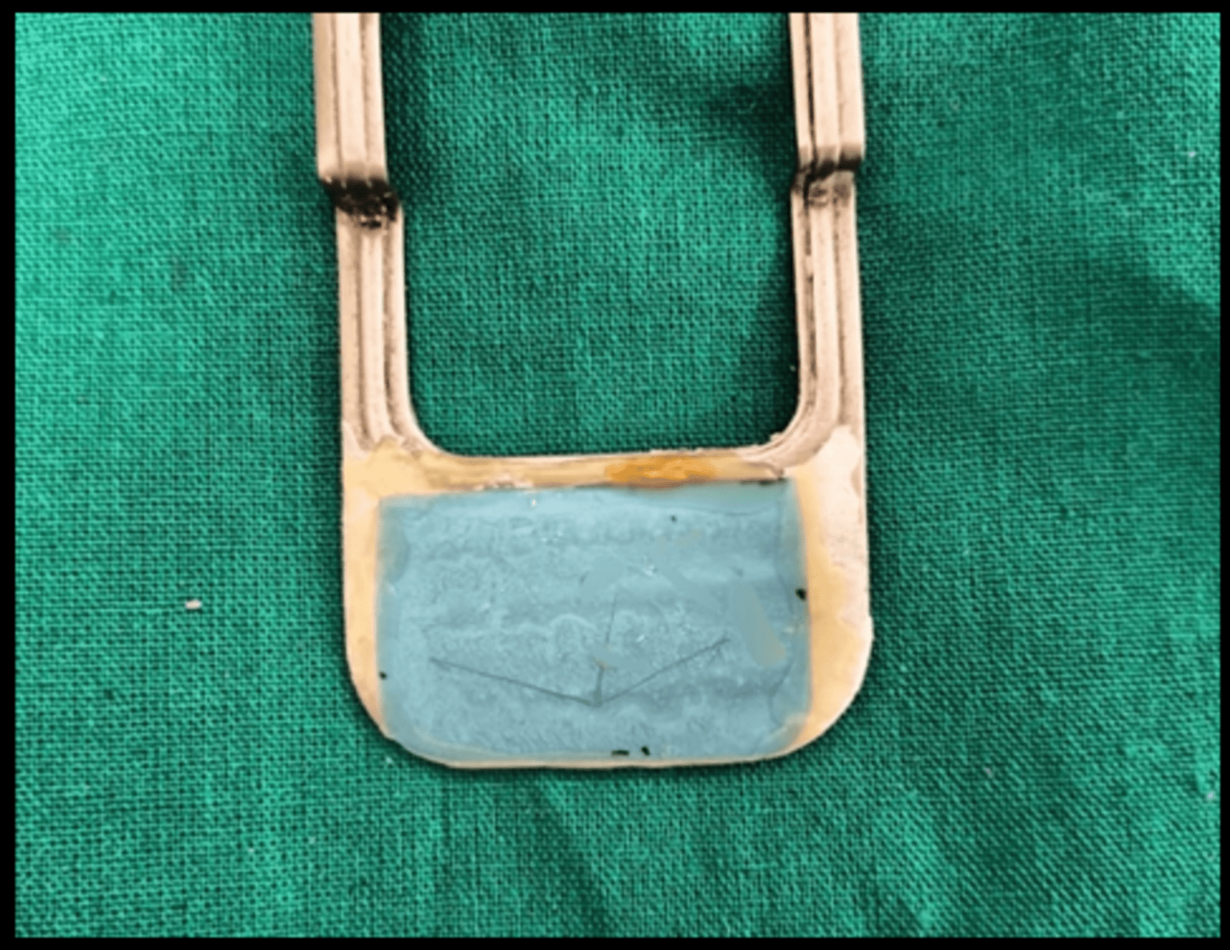
Arrow point tracing

Right after the cast is oriented on the articulator, the extra-oral height tracing device is attached to the rims, and the patients are trained to trace of the mandibular border movements as given in the Ney mandibular excursion guide. A point is drawn 6 mm from the peak of the trace on the clear sheet to show the protrusive position (Figure 3). Afterward, a small circular nick is made on the surface of both occlusal rims in the molar and canine regions, and it is coated with petrolatum for ease of separation of the recording material, and with the interocclusal recording material, the records are obtained. We have used the polyvinyl siloxane material (Jet Bites), the bite registration pastes, and bite registration wax (reinforced wax, ALUWAX) as an interocclusal recording material. For recording the centric relation record, the patient is asked to bite gently over the interocclusal recording material, and for protrusive records, the patient is asked to close the lower jaw in a forward position such that the stylus is positioned on the 6 mm marking on the tracing plate. The interocclusal materials are then used to record it. 

**Figure 4:**
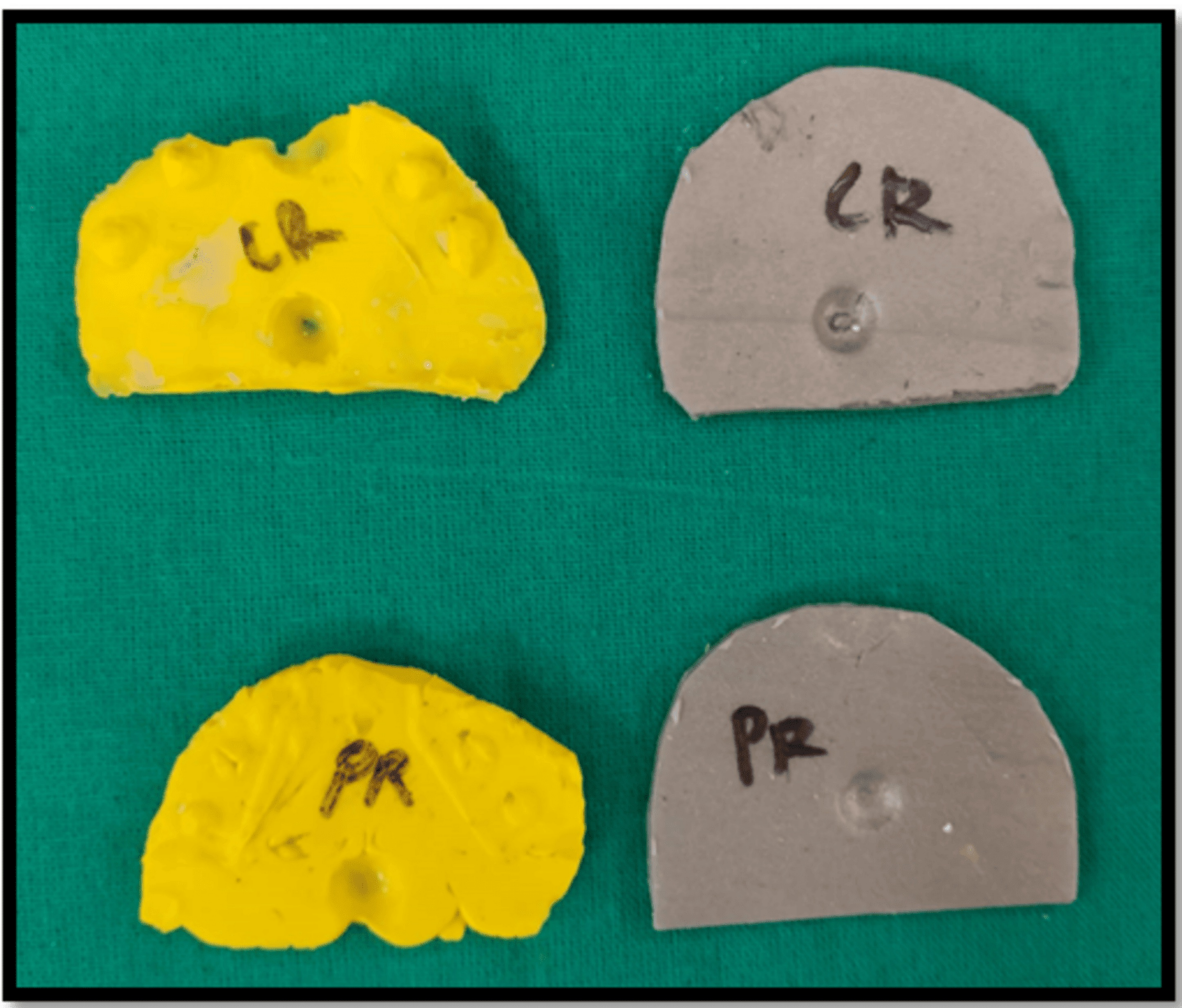
Interocclusal records of bite registration paste and reinforced wax CR: Centric record, PR: Protrusive record

Once the records have been obtained (Figure 4), using Lauritzen's split cast technique, the articulator is programmed. With the centric record in place, the cast was oriented and articulated in the exact centric position. Also, by using the protrusive record, the articulator is programmed to determine the HCI. Once the required HCI is determined, the Bennett angle is obtained using Hanau’s formula L = H/8 + 12. This data was then subjected to statistical analysis by Pearson's correlation.

## Results

The values obtained from OPG were 10-20° greater than anatomic values on average. The angle is formed by the intersection of the horizontal reference line and mean curvature line, which is obtained by tracing the outline of articular eminence and FHP for each side to get an HCI value that is compared with the values obtained after programming the articulator with different materials. The correlation analysis yields the results as, for the seven participants, there was a substantial association between the angles discovered in the articulator and the associated radiography image. The mathematical evaluation by Pearson correlation value (r) for articulator value on the right side with bite registration wax material versus OPG is 0.844 at a significant level of 0.01 (two-tailed) and with bite registration paste material versus OPG value is 0.885 at a significant level of 0.01 (two-tailed) (Table 1).

**Table 1:** The relationship between the angle obtained from the articulator and OPG on the right side n: number of participants, r: Pearson's correlation, ** Pearson’s correlation, Correlation is significant at the 0.01 level (2-tailed), OPG: Orthopantogram

Condylar Inclination	n	r	Significant
Articulator Right for bite registration wax verses Opg right	7	.844**	0.01
Articulator right for bite registration paste verses Opg right	7	.885**	0.01

In this study, the Pearson correlation coefficient (r) for the left-side angle between the articulator and the OPG was found to be 0.725 when using bite registration wax material. On the other hand, the correlation coefficient between the articulator and the OPG was slightly higher at 0.762 when using bite registration paste material. Both of these correlations were statistically significant at the 0.05 level in a two-tailed test (Table 2). These results suggest that the angle obtained from the articulator when using bite registration paste material is more closely aligned with the angle measured by tracing, as indicated by the stronger correlation with the OPG. Consequently, the angle obtained from the articulator versus the OPG shows a more direct relationship with the bite registration paste compared to the wax material.

**Table 2:** The relationship between the angle obtained from the articulator and OPG on the left side n: number of participants, r: Pearson's correlation, *Pearson’s correlation, Correlation is significant at the 0.05 level (2-tailed), OPG: Orthopantogram

Condylar Inclination	n	r	Significant
Articulator left for bite registration wax verses Opg left	7	.725	0.00
Articulator left for bite registration paste verses Opg left	7	.762*	0.05

In the descriptive analysis, the study included seven respondents to calculate the mean values for the angles on both the left and right sides using the articulator. These calculations provided a comprehensive understanding of how closely the articulator’s measurements align with those obtained from the OPG, further emphasizing the effectiveness of the bite registration paste in achieving more accurate results. For the bite registration, paste the mean value of the angle on the left side of the articulator, which is 18.428, the standard deviation (SD) value is 4.076, and the standard error (SE) is 1.573. The mean value of the angle on the left side for bite registration wax is 22.714, the SD value is 5.089, and the SE is 1.92372. Condylar inclination in OPG: the mean value for the left side of condylar inclination is 29.2857, the SD is 3.81725, and the SE is 1.44279 (Table 3).

**Table 3:** The mean value angles, SD between the horizontal reference line and mean curvature line on the OPG and on the articulator left side with bite registration paste and with bite registration wax material SD: standard deviation, SE: standard error, n: number, OPG: orthopantogram

Inclination of Condyle	Mean value	SD	SE	n
Bite registration paste value on left side of articulator	18.428	4.076	1.573	7
Bite registration wax value on left side of articulator	22.714	5.089	1.923	7
OPG left	29.285	3.817	1.442	7

The following results are obtained from the descriptive analysis. The seven respondents are put in this study for calculation; for the bite registration paste, the mean value of the angle measured on the right side of the articulator was found to be 21.000 degrees. The SD, which indicates the amount of variation or dispersion from the mean, was 4.163 degrees. The SE, which represents the accuracy with which the sample mean estimates the population mean, was consistent with the SE value observed on the left side. In contrast, when using bite registration wax, the mean value of the angle on the right side of the articulator was higher, at 25.714 degrees. The standard deviation in this case was 5.122 degrees, suggesting a slightly greater variation around the mean compared to the paste. The standard error for the wax material was 1.936. Additionally, measurements taken from the OPG revealed that the average condylar inclination was 31.428 degrees. The standard deviation for this measurement was 4.117 degrees, reflecting a degree of variability similar to that observed with the bite registration paste. The standard error associated with the OPG measurement was 1.331, indicating the reliability of the mean condylar inclination value derived from the sample. These results, as detailed in Table 4, provide a comprehensive comparison between the bite registration paste and wax materials concerning their ability to accurately reflect the angles of condylar inclination when measured on the right side of the articulator and compared to the OPG. This adds clarity and expands on the statistical results, emphasizing the comparison between materials and the significance of the findings.

**Table 4:** The mean angles, SD between the horizontal reference line and mean curvature line on the OPG and on the articulator right side with bite registration paste and with bite registration wax material SD: standard deviation, SE: standard error, n: number, OPG: orthopantogram

Inclination of Condyle	Mean value	SD	SE	n
Bite registration paste value on right side of articulator	21.000	4.163	1.573	7
Bite registration wax value on right side of articulator	25.714	5.122	1.936	7
OPG right	31.428	4.117	1.556	7

## Discussion

The most important aspect of making a complete denture prosthesis is to achieve harmony with the individual's stomatognathic system [8]. The importance of condylar guidance in creating a dental prosthesis is of utmost significance as it represents the course of the mandibular condyle along the TMJ. The angle of the condylar guidance affects mandibular movements, ensuring proper tooth contact during chewing and speaking. Accurate reproduction of condylar guidance facilitates efficient mandibular movements, enhancing masticatory efficiency and comfort. This precision reduces the risk of temporomandibular joint disorders by promoting smooth, uninterrupted condyle movements within the joint, preventing abnormal stresses that could cause TMJ pain and dysfunction. At the step of jaw relation, establishing accurate condylar guidance is essential to achieve a balanced occlusion, which reduces occlusal interferences and ensures even force distribution across dental arches, thus minimizing stress on individual teeth. By accurately capturing the protrusive or lateral movements of the mandible, clinicians can ensure that the articulator replicates these movements, allowing for more precise and customized prostheses [9]. Effective prosthodontic practice can be enhanced by precisely replicating the path of movements of the condylar head over the articular eminence on a semi-adjustable articulator [10,11]. In 1902, Christensen proposed using a protrusive interocclusal record to program the semi-adjustable articulator. In 1930, Hanau introduced a formula, L = H/8 + 12, to calculate lateral condylar guidance for the same purpose [12].

There are various techniques to record condylar guidance, such as extraoral and intraoral tracing devices, by interpretation of radiographs with intraoral recording materials [13]. In our study, we have used two methods to obtain CG: one by interpretation of a radiograph, which is kept as standard values, and the other value obtained from interocclusal records. A comparison is then done between the two methods. The method for obtaining the values by radiograph includes tracing the articular eminence inclination in the OPG from the most superior to the most inferior points of curvature, showing the curve's mean inclination. This could differ from directing inclination with about 4-6 mm of protrusion, which is a clinically important range of protrusion and CG [14,15]. Regulated radiation exposure at appropriate intervals ensures no image overlap and proper radiography on the film. An OPG provides a complete image of the maxilla, mandible, and surrounding structures on a single film, though its accuracy is limited by picture distortion and magnification. White and Pharaoh et al. proposed that consistent horizontal and vertical magnification in the focal trough's middle plane would ensure all structures are in focus on the final radiograph [16-19].

In semi-adjustable articulators, the inclination of CG is programmed either by intraoral or extraoral tracers, which provide the lateral or protrusive record [20]. McCollum and Gysi introduced and demonstrated extraoral tracing methods, which were utilized to record condylar guidance in totally edentulous patients [21]. The Gothic arch tracing technique consistently records jaw movements, which are then programmed on a semi-adjustable articulator to facilitate the routine denture prosthesis fabrication process [22,23]. The Interocclusal records are thought to be the best technique to program the articulator. Choosing the correct bite registration material is crucial for capturing the precise relationship between maxilla and mandible, which is essential to creating well-fitting and functional dentures. Modern bite registration materials are designed to set quickly and comfortably, minimizing the time patients need to keep their mouths open and enhancing their experience. These materials must maintain dimensional stability over time to ensure accuracy from the moment of recording to laboratory use. Versatile options such as pastes, putties, acrylic resin, thermoplastic polymers, and wafers allow dentists to select the most appropriate material for each patient, ensuring optimal results [9]. High-quality bite registration materials led to better clinical outcomes, providing edentulous patients with comfortable and functional dentures that significantly improve their quality of life. In this study, we have used reinforced wax and jet bite.

Reinforced wax and polyvinyl siloxane are highly researched materials for bite registration in denture fabrication due to their properties that enhance accuracy and efficiency. Reinforced wax offers improved dimensional stability, moldability, and resistance to tearing, ensuring precise occlusal relationships and preventing distortions over time. The precision of these waxes depends on their quality and processing, although uneven softening and thickness can pose challenges [24]. Jet Bite, a polyvinyl siloxane material, is favored for its fast-setting time, enhancing patient comfort and cooperation. It accurately reproduces fine occlusal details and maintains accuracy with excellent elastic recovery during handling and transport. Additionally, Jet Bite is easy to handle and apply, simplifying the bite registration process and ensuring well-fitting, functional dentures [25]. Research on these materials focuses on comparing their effectiveness with traditional methods and evaluating improvements in accuracy, patient comfort, and overall outcomes in denture fabrication. The goal is to determine how reinforced wax and jet bite can enhance the reliability and efficiency of bite registrations, ultimately leading to better-fitting, more comfortable dentures for edentulous patients.

Silicone-based bite registration materials are ideal for capturing accurate occlusal relationships in dental procedures due to their excellent dimensional stability, high accuracy, and ability to capture fine details. Their fast-setting time and smooth consistency enhance patient comfort and cooperation. These materials offer excellent elastic recovery, ensuring accuracy during handling and transportation. Easy to handle and often supplied in auto-mix cartridges. Despite the quick setting, they provide sufficient working time for adjustments and are compatible with other dental materials. Durable and resistant to tearing, silicone-based materials maintain the integrity of the bite registration, making them an excellent choice for precise, reliable, and comfortable results. According to the authors Hsu and Millstein, the interocclusal record is expected to be more dimensionally stable and to provide a detailed representation of an interocclusal space that is later used to program the semi-adjustable articulator [26].

In this study, there was a strong association found amongst the seven patients between the angle obtained on the articulator by two different interocclusal recording materials and the orthopantomogram. The statistical analysis with the Pearson correlation test was performed at a 99% confidence interval. It shows the stronger correlation value for bite registration paste material; the value of articulator versus OPG on the left and right sides, respectively, is 0.762 and 0.885. For bite registration wax material, the value of the articulator versus OPG is 0.725 and 0.844 on the left and right sides, respectively. This proves that the angle obtained with bite registration paste material on the articulator is closely correlated with that obtained from OPG. The current study concluded that polyvinyl siloxane material provides a more reliable value than reinforced wax compared to the angle obtained by tracing the outline on OPG. In our study, we found that the average interocclusal registration value of condylar guiding inclination for bite registration wax ranges from 13° to 23° and for bite registration paste ranges from 15° to 28°. According to Preti et al., the average interocclusal registration values for condylar guiding inclination range from 21° to 64° [27].

Our study found that in the radiographic technique produced, an LCG value was higher than the value calculated using Hanau’s formula; this was also supported in the literature [28]. We also noted the difference in values of right and left condylar guidance, which is supported in the literature by El-Gheriani et al., who reported variances in right and left values of CG [29]. Boucher suggests achieving a centric relationship with minimal pressure to ensure homogeneous contact of the denture bases; waxes might fail to record [30]. Vassilis K et al. evaluated four materials: acrylic resin, polyether, wax, and polyvinyl siloxane for their efficacy in recording vertical interocclusal relationships, finding that polyvinyl siloxane and polyether had the lowest discrepancies (24-74 μm), while wax had the highest, which supports our finding that polyvinyl siloxane material is more reliable than waxes [31]. Gilboa and Harold's study assessed the correlation between the anatomy of articular eminence and equivalent OPG images in dry human skulls. They found that OPG images reliably replicated the inclinations of the articular eminences, with a difference of only seven degrees compared to 25 human skulls [32]. In our study, we found an average difference of 10° between the angles obtained from OPG and those acquired from an articulator for polyvinyl siloxane material. And with bite registration wax, the mean difference noted was seven degrees. The average inaccuracy between the right and left sides of the articulator and OPG for bite registration paste is 1.5° and for bite registration wax is 1.9°.

Though the study has encountered some limitations, such as that it cannot be performed on patients who lack neuromuscular coordination, Anatomical and physiological variability among subjects affecting measurement accuracy; and inherent limitations in the precision of digital panoramic radiographs. Environmental factors like temperature and humidity may further influence material properties. These limitations were taken into consideration before starting the study and taken care of. Acknowledging these limitations is crucial for enhancing the study's credibility and guiding future research directions.

## Conclusions

This study shows a pivotal step towards assessing the effectiveness of the interocclusal record material used in conventionally used Gothic arch tracing techniques. By leveraging advancements in digital technologies, particularly in the realm of prosthodontics, we can potentially discover more reliable techniques for diagnosis and treatment planning. The evolution of digital technologies offers the promise of providing more precise tools for these purposes. This approach has the potential to minimize errors and streamline the process of achieving harmonized occlusion, thereby optimizing treatment outcomes while saving valuable time.
